# Host-Microbe Interactions in Airway Disease: toward Disease Mechanisms and Novel Therapeutic Strategies

**DOI:** 10.1128/mSystems.00158-17

**Published:** 2018-03-13

**Authors:** Emily K. Cope

**Affiliations:** a Department of Biological Sciences, Pathogen and Microbiome Institute, Northern Arizona University, Flagstaff, Arizona, USA

**Keywords:** airway microbiome, asthma, chronic rhinosinusitis, host-microbiome interaction, microbial therapeutics, probiotics, respiratory microbiome, sinusitis

## Abstract

Despite growing efforts to understand the role of the microbiota in airway disease, mechanisms that link microbial community dysbiosis to chronic inflammation remain elusive. Our laboratory is interested in how altered microbiota composition or function influences airway inflammatory diseases, including chronic rhinosinusitis, asthma, and cystic fibrosis. Given the tight interplay between host-associated microbes and host immunity, the potential for translational microbiome research to guide clinical decisions and novel therapeutics is becoming better appreciated. We hope to advance our understanding of the ecology of airway disease through integrating multiple omics assays and *in vitro* and *in vivo* experimental validation. An increased understanding of the role of the microbiota in chronic airway inflammation will ultimately lead to the rational development of therapeutics aimed at manipulation of microbiota composition or activity to treat these important and costly diseases. In this perspective, I discuss our current research investigating the microbiology and ecology of the airway microbiome.

## Abstract (precis)

Despite growing efforts to understand the role of the microbiota in airway disease, mechanisms that link microbial community dysbiosis to chronic inflammation remain elusive. Our laboratory is interested in how altered microbiota composition or function influences airway inflammatory diseases, including chronic rhinosinusitis, asthma, and cystic fibrosis.

## PERSPECTIVE

Translational microbiome research has generated a wealth of data associating altered microbial communities with diseases of the gastrointestinal (GI) tract (1), skin (2), and respiratory tract (3, 4) and even cognitive and neurological disorders (5). Mechanistic studies, which have focused predominantly on the gut, have led to the identification of potential avenues for microbiome-directed therapeutics. For example, murine studies indicate that microbe-derived short-chain fatty acids can induce colonic FOXP3^+^ CD4^+^ regulatory T-cell proliferation via epigenetic modification (1) and alter dendritic-cell function through activation of G protein-coupled receptors GPR43 and GPR41 (6). Whether these observations made in animals are relevant in diseases where extensive interpersonal variation in the microbiome and immune response exists is unknown. Below, I discuss two of the primary goals in my laboratory to advance the field of human microbiome research in the context of airway diseases, specifically, chronic rhinosinusitis (CRS), asthma, and cystic fibrosis (CF), performed in close collaboration with clinical partners. First, we aim to use a multiple-omics approach (integrating metagenomics, metatranscriptomics, and metabolomics/metaproteomics) to understand the composition and function of the host-associated airway microbiota. Our second goal is to identify microbiota-derived molecules that promote the selection of pathogenic microbial communities and drive specific inflammatory responses. Ultimately, we aim to understand human host-microbiome ecology to inform the development of microbiome-directed therapeutics to treat or prevent airway inflammation.

## AIRWAY DISEASE AND LOCAL MUCOSAL MICROBIOME

An emerging body of evidence indicates that local mucosal microbial composition and function are related to the host immune response in the airways (3) and at other mucosal sites (7). Our group is interested in chronic inflammatory diseases of the airways, including CRS, a disorder of the sinonasal cavity characterized by inflammation lasting >12 weeks. Epidemiological surveys suggest that CRS costs up to $65 billion each year and affects approximately 16% of the U.S. population (8). Not only is CRS one of the most prevalent chronic diseases in developed countries, CRS patients report a significantly worse quality of life than those with asthma, congestive heart failure, and chronic back pain (9). Despite the significant socioeconomic impact, the underlying cause of sinonasal inflammation is not well understood.

CRS often exists in a setting of concomitant lower airway disease (10). Two examples of this connection, CF-associated CRS (CF-CRS) and asthma-associated CRS, provide an opportunity to further characterize the relationship between the microbiota and immune response across the sinonasal cavity and lungs in the context of the “unified airway hypothesis,” which posits that concurrent sinonasal and pulmonary diseases are manifestations of a single inflammatory process. Altered microbial communities have been reported in the sinonasal cavities of CRS patients (3) and in the lungs of asthmatics and CF patients (4); however, the microbiomes or transcriptomes of these sites have not been examined in parallel. We are using a multiomics approach to investigate paired sinonasal and lung specimens from CRS patients with pulmonary comorbidities to determine whether key microbes or microbial functions correlate with concurrent inflammation to generate hypotheses regarding which key microbes or microbial products drive inflammation. Our preliminary results indicate that while CRS patients without clinically diagnosed pulmonary disease exhibit site-specific microbial communities in their sinuses and lungs, CF-CRS patients do not, suggesting microbial translocation between these two sites in patients with impaired mucociliary clearance and immune response impairment.

Disease heterogeneity is an issue when diagnosing and treating CRS; our recent work has demonstrated high interpersonal variability in CRS patient microbiotas compared to specimens from the same patient (11). We have identified four distinct sinus microbiota compositions in CRS patients, each dominated by a distinct respiratory pathogen that coassociates with a specific pattern of low-abundance bacteria. Each of these pathogenic communities associates with a distinct mucosal immune response (3). On the basis of these fundamental observations, the first goal of my laboratory is to determine how the mucosal microbiota drives inflammatory responses in these patients to understand the basis of patient heterogeneity and derive therapeutics specific to the microbial and immune dysfunctions in patient subgroups. Integrating multiple omics tools to characterize host and microbiome features of large, longitudinally collected cohorts will be essential to move from correlation to causation by informing specific hypotheses that can be tested *in vitro* and in disease models. Layering of multiomics assays will permit identification of the primary microbial producers and the molecules that mediate microbiome-host interactions underlying chronic inflammation. The resolution achieved through metagenomic sequencing will capture the functional genomic potential and strain level diversity of bacteria, fungi, and viruses in patients with CRS. The power of this approach was recently demonstrated in a study of atopic dermatitis (2). Clonal populations of *Staphylococcus aureus* were associated with more severe disease in humans; distinct strains of *S. aureus* and *S. epidermidis* conferred differences in murine Th2 and Th17 cell expansion and epidermal thickening (2). I anticipate that, as the cost of high-throughput sequencing and mass spectrometry continues to decline, functional metaomics will become more accessible on limited scientific and clinical budgets and will play a central role in advancing our understanding of the role of the microbiota in human disease.

A second goal of my laboratory is to identify microbiota-derived molecules that promote the selection of pathogenic microbial communities and drive specific inflammatory profiles. Bacteria and fungi coinhabit the human body, and the microbial interactions within niche-specific microbiomes can influence health and disease. Bacterial cocolonizers can directly affect fungal morphology, growth, virulence, and attachment to host epithelia or other microbes (12). We are interested in how cocolonization patterns observed in our human CRS cohorts are maintained or selected for and whether these patterns change over time within the course of single or multiple sinusitis exacerbations. Because mucosa-associated microbiotas in the sinuses exist predominantly as biofilms, a major component of our research is to determine whether microbial communication via signaling molecules can influence pathogenicity and dictate the host immune response. Microbial metabolites and quorum-sensing molecules are important mediators of microbial communication that permit microbes to respond to environmental stimuli. Future studies that consider the importance of microbial signaling in the human microbiome may lead to novel therapeutics aimed toward manipulating the relative abundances of microbial species in a niche. For example, introduction of an engineered strain of *Escherichia coli* that produces excess AI-2, an interspecies signaling molecule, can shape the composition of the microbiota by impeding the expansion of *Bacteroides* and promoting the growth of *Firmicutes* (13). Recent work with *ex vivo* cell populations has identified specific metabolites (12,13-DiHOME) that influence dendritic-cell–T-cell interactions, leading to suppression of anti-inflammatory Foxp3^+^ CD25^+^ CD4^+^ Treg cells (7). Future studies using patient-derived cell populations and representative models of mucosal surfaces will be crucial to infer causality. Microfluidic devices such as the HuMiX system that recapitulates the oxygen gradient, mucin layer, and immune systems at mucosal surfaces will prove useful tools for understanding host-microbe interactions (14). Other biomimetic microsystems that reproduce key structural and mechanistic properties of organs may also gain traction in future microbiome studies to screen for potential pre- or probiotics prior to costly clinical trials. Mechanistic studies that isolate microbe-microbe-host interactions will yield important insights that will allow us to judiciously design therapeutics that incorporate appropriate microbial signals with protective microbial taxa to restore microbial and host health.

## FUTURE WORK

Given the tight interplay between the microbiota and host immunity, the potential for microbiome research to guide clinical decisions and novel therapeutics is becoming better appreciated. Advances in our understanding of the microbial mechanisms that drive disease and maintain host immune homeostasis will facilitate the development of strategies for microbiome manipulation, which holds great potential for treating a myriad of human diseases. I anticipate that the next 5 years will bring advances in our understanding of the ecology of airway disease through a systems biology approach facilitated by the application of layered multiomics paired with experimental validation.

Like other translational microbiome projects, we hope to understand how dysbiotic microbiotas drive inflammation and develop strategies to manipulate these communities. At this juncture, we should use caution when developing therapeutics designed to manipulate microbiotas before we fully understand the mechanisms driving disease and microbial interactions. Premature efforts could lead to ineffective treatment because we do not yet understand the complexity of microbial interactions that could lead to increased host inflammatory responses or virulence. Microbiotas in peripheral organs, such as the gut, may also influence airway health. Experimental evidence suggests that manipulation of the GI microbiota can alleviate or prevent allergic airway disease (6). However, there have been no such investigations to determine the influence of diet on the sinonasal microbiota and the development of CRS. We are interested in determining whether dietary manipulation can reduce Th2 inflammation in CRS patients.

As a field, we should develop and embrace multiomics approaches that integrate information from metagenomes, metabolomes, and metatranscriptomes to obtain a more comprehensive understanding of the microbiological and ecological processes that govern microbial community function within a niche. This will inform our design of future microbiota-targeted therapeutics, including whether keystone or foundation species are appropriate, whether the goal of probiotics should be stable engraftment or transient colonization of a niche, and whether a single species or a consortium of taxa should be transferred to treat disease.
